# Effect of Annealing on the Structural, Magnetic and Surface Energy of CoFeBY Films on Si (100) Substrate

**DOI:** 10.3390/ma14040987

**Published:** 2021-02-19

**Authors:** Wen-Jen Liu, Yung-Huang Chang, Yuan-Tsung Chen, Yi-Chen Chiang, Yu-Chi Liu, Te-Ho Wu, Po-Wei Chi

**Affiliations:** 1 Department of Materials Science and Engineering, I-Shou University, Kaohsiung 840, Taiwan; jurgen@isu.edu.tw; 2 Bachelor Program in Industrial Technology, National Yunlin University of Science and Technology, 123 University Road, Section 3, Douliou, Yunlin 64002, Taiwan; g9213752@gmail.com; 3 Graduate School of Materials Science, National Yunlin University of Science and Technology, 123 University Road, Section 3, Douliou, Yunlin 64002, Taiwan; M10947001@yuntech.edu.tw (Y.-C.C.); M10947009@yuntech.edu.tw (Y.-C.L.); wuth@yuntech.edu.tw (T.-H.W.); 4 Institute of Physics, Academia Sinica, Nankang, Taipei 11529, Taiwan; jacky01234567891@hotmail.com

**Keywords:** annealed Co_40_Fe_40_B_10_Y_10_ thin films, low-frequency alternating current magnetic susceptibility (χ_ac_), optimal resonance frequency (f_res_), surface energy, adhesion, transmittance

## Abstract

The structure, magnetic properties, optical properties and adhesion efficiency of CoFeBY films were studied. Co_40_Fe_40_B_10_Y_10_ alloy was sputtered onto Si (100) with a thickness of 10–50 nm, and then annealed at room temperature, 100 °C, 200 °C and 300 °C for 1 h. X-ray diffraction (XRD) showed that the CoFeBY films deposited at room temperature are amorphous. Annealing at 100 °C gave the films enough thermal energy to change the structure from amorphous to crystalline. After annealing, the CoFeBY thin film showed a body-centered cubic (BCC) CoFeB (110) characteristic peak at 44°. However, the low-frequency alternative-current magnetic susceptibility (χ_ac_) and saturation magnetization (M_S_) increased with the increase of thickness. CoFeBY thin films had the highest χ_ac_ and M_S_ after annealing at 300 °C compared to that at other temperatures. After annealing at 300 °C, the surface energy of CoFeBY film is the maximum at 50 nm. Higher surface energy indicated stronger adhesion.

## 1. Introduction

Magnetic nanomaterials have attracted more and more attention in recent years owing to their unique magnetism and potential applications in data storage. Among the magnetic metals, the CoFeB alloy is a kind of soft magnetic material with high saturation magnetization (M_S_) and low coercivity (H_c_). The CoFeB has received much attention; the development of its ferromagnetic (FM)/antiferromagnetic (AFM) exchange-biasing anisotropy has increased the tunneling magnetoresistance (TMR) and enhanced the magnetic anisotropy of the structure [1,2,3,4,5]. The CoFeB thin film can also be used as a free layer or a pinned layer to form a magnetic tunnel junction (MTJ) with the MgO layer. In addition, CoFeB materials also attracted special attention in different applications. Among other uses, sensor applications of the materials in which induced magnetic anisotropy occurs are very important. It was shown that stress of field induced magnetic anisotropy can be formed in such materials and their high frequency applications are demonstrated [6,7]. The annealed CoFeB film exhibits B diffusion and develops perpendicular magnetic anisotropy [8,9,10,11]. Therefore, CoFeB thin films can be used as potential materials for magnetoresistance random access memory (MRAM), read/write heads and hard disk devices. The magnetic properties of a material are influenced by its thickness, shape, crystallinity and interface interaction. Often, improvements in TMR values of the CoFeB/MgO structure are required to improve TMR ratios. When the crystallization temperature is higher than that of the sample, the nanocrystalline structure exhibits excellent soft magnetic properties, which is related to the strong intergranular magnetic exchange mediated by a ferromagnetic amorphous matrix. The tunneling magnetoresistance (TMR) of CoFeB-type MTJ changes when the amorphous state changes to nanocrystalline. However, annealing at 350 °C or higher not only reduces the TMR ratios but also causes the loss of magnetic properties due to an excessively high annealing temperature [12,13,14]. Therefore, the magnetic characteristics of CoFeB films at room temperature (RT) or in an annealing environment have been the focus of several studies. Rare earth elements have very unique characteristics that can be used to improve the high-temperature resistance, including their mechanical strength and other physical properties of magnetic films. Perhaps the rare earth elements can be used to enhance and solve the thermal stability of magnetic films at high temperatures. The element yttrium (Y) has a high abundance in rare earth ore, but it has been much less utilized. The addition of Y to the alloy matrix and heat treatment significantly improves mechanical properties such as thermal stability and corrosion resistance [15]. Y substitution can also enhance the exchange coupling effect, which not only improves the remanence and magnetic energy of the product, but also improves the thermal stability of the product [16,17,18,19]. When Y is added to soft magnetic materials, such as Fe-Y-B alloy, it is necessary to increase the soft magnetic performance through some methods such as low-temperature annealing, because Y has hard magnetic properties [20,21,22]. However, the effect of the addition of Y to CoFeB is not fully documented. It would be interesting to study the effect of the addition of the rare earth element Y to the CoFeB alloy, perform thermal annealing and examine the magnetic properties of the CoFeB film. Therefore, it is great significance to investigate the characteristics of CoFeBY films deposited by sputtering technique through RT and post-annealing treatments. The different thickness (t_f_) of as-deposited and annealed CoFeBY films were also studied and we investigated the effects of crystallinity on the magnetic properties, adhesion and optical performance of the films. In our previous study, the properties of as-deposited Glass/CoFeBY and Si (100)/CoFeBY films were studied, as shown in Table 1 [23,24]. Continuing forward, we studied the as-deposited and annealed CoFeBY thin films. We found that the low-frequency alternate-current magnetic susceptibility (χ_ac_) ranges of CoFeBY films were higher than those of other Glass/CoFeVB and Si (100)/CoFeVB materials.

## 2. Materials and Methods 

CoFeBY thin films with a thickness of 10–50 nm were sputtered onto Si (100) substrates by direct current (DC) magnetron sputtering at room temperature (RT). The films were prepared under the following four conditions: (a) at room temperature, (b) annealed at 100 °C for 1 h, (c) annealed at 200 °C for 1 h and annealed at 300 °C for 1 h. The power density was 1.65 W/cm^2^ and the deposition rate was 1.2 nm/min. The chamber base pressure was 3 × 10^−7^ Torr, and the Ar working pressure was 3 × 10^−3^ Torr. The pressure in an ex-situ annealed condition was 2.5 × 10^−3^ Torr with a specific Ar gas. The alloy target for the composition of CoFeBY was 40 at% Co, 40 at% Fe, 10 at% B and 10 at% Y. The grazing incidence X-ray diffraction (GIXRD) patterns of Cukα1 (PAN analytical X’pert PRO MRD, Malvern Panalytical Ltd, Cambridge, UK) and low angle diffraction incidence of about 2° were used to determine the structure of CoFeBY films. The in-plane low-frequency alternate-current magnetic susceptibility (χ_ac_) and hysteresis loop of Co_40_Fe_40_B_10_Y_10_ films have been studied by using χ_ac_ analyzer (XacQuan, MagQu Co. Ltd., New Taipei City, Taiwan) and alternating gradient magnetometer (AGM, PMC, Ohio, OH, USA). The standard sample was calibrated by external magnetic field χ_ac_ measurement. The sample was inserted into the χ_ac_ analyzer. The range of driving frequency was between 10 and 25,000 Hz. χ_ac_ was determined by magnetization. All specimens had the same shape and size to eliminate demagnetization. The χ_ac_ valve is an arbitrary unit (a.u.) which due to exchange result corresponded to the reference standard sample, which is a comparative value. The relationship between magnetic susceptibility and frequency was measured by the χ_ac_ measurement. The optimal resonance frequency (f_res_) was detected by χ_ac_ analyzer, which reveals the frequency of the maximum χ_ac_. The contact angle of CoFeBY film was measured with deionized water and glycerol. The accuracy of each contact angles is measured by three times. The contact angle was an averaged value. The surface energy was obtained from the contact angle and some calculations [25,26,27].

## 3. Results

### 3.1. Structure Property and Grain Size Distribution

**Figure 1:**
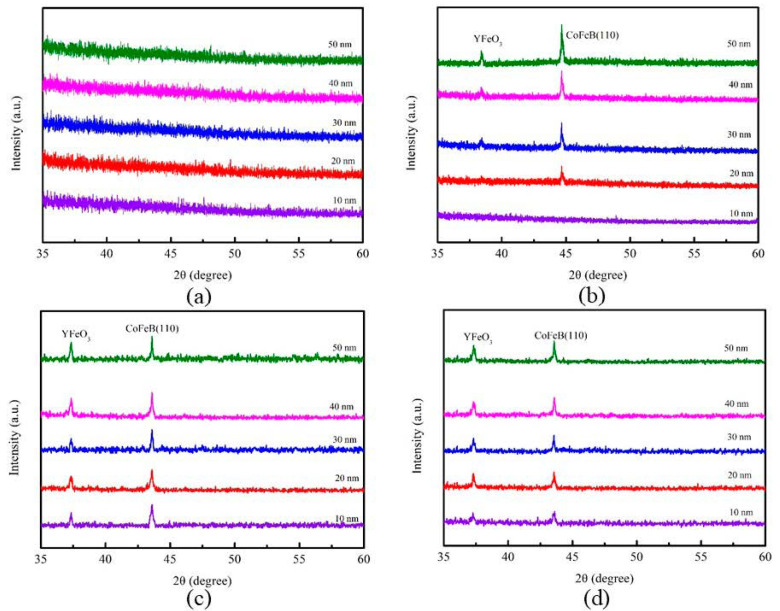
X-ray diffraction (XRD) patterns of CoFeBY films. (**a**) room temperature (RT), (**b**) after annealing at 100 °C, (**c**) after annealing at 200 °C, (**d**) after annealing at 300 °C.

Figure 1a–d show the X-ray diffraction (XRD) pattern of the as-deposited and annealed CoFeBY thin films.

The Si (100) substrate used in this study had strong diffraction peaks at 33.2° and 70°; therefore, the XRD was displayed at a diffraction angle (2θ) between 35 and 60 degrees. There was no obvious crystallization peak, indicating the amorphousness of thin films deposited at RT and annealed 100 °C at 10 nm thickness. Therefore, the thickness of the CoFeBY thin films may be considered as a discontinuous growth model where random atomic arrangements lead to an amorphous state. At an annealing temperature of 100 °C and 20 nm thickness, the structure could transform from an amorphous state to a crystalline state. XRD intensity data shows the characteristic peak at around 2θ = 44°, as shown in Figure 1b–d, which is attributed to the CoFeB with body-centered-cubic structure (110) orientation. Further annealing at temperatures higher than 100 °C relieved stresses and caused a loss of tensile stresses, as is obvious from the slight shift of the CoFeB peak to the left, attributed to the annealing effect. Bragg’s law applied due to the expansion of lattice parameters, resulting in the replacement of larger atoms in the matrix. This indicates that the lattice parameter expands due to the inclusion of Y atoms. Although the chamber was pumped to 10^±7^ Torr in the sputtering system, oxygen may still be present. Both natural oxides on Si (100) substrate and oxygen contamination on a sputtering target contributed to the formation of YFeO_3_. The full width at half maximum (FWHM) measured by XRD was used to calculate the grain size by the Scherrer equation. 

Scherrer′s formula (Equation (1)) is [28],

D = kλ/βcosθ(1)

**Figure 2:**
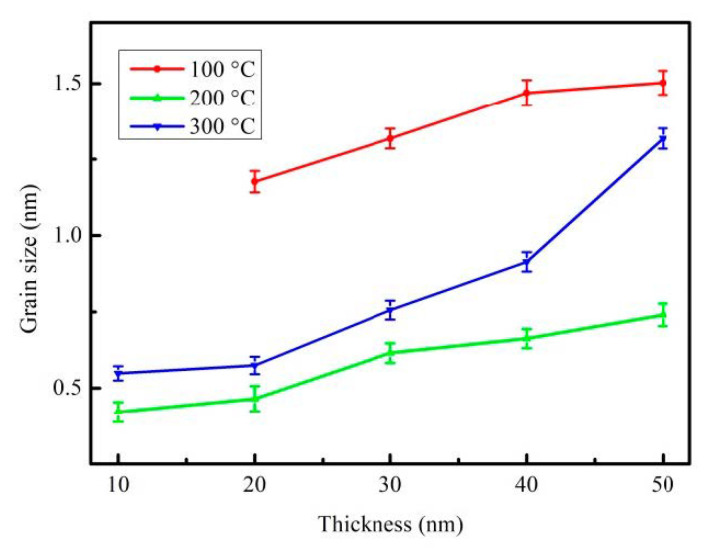
The mean grain size distribution of CoFeBY films.

In the formula, k (0.89) denotes the Scherrer′s constant; λ represents the X-ray wavelength of the Cu Kα1 line, B is the FWHM diffraction CoFeB (110) peak and θ is the half-angle of the diffraction peak. The mean grain sizes estimated from the half maximum (FWHM, B) of the CoFeB (110) peak under four annealed conditions are plotted in Figure 2. The observed grain size, the CoFeBY thin film that had been post-annealed at 100 °C, possessed a bigger grain size, and the film that had been annealed at 200 °C possessed a smaller grain size. Furthermore, grain sizes following annealing at 200 °C and 300 °C were smaller than that post-annealed at 100 °C. This because on adding a small amount of Y to the alloy, the grains become smaller after annealing due to grain refinement [29].

### 3.2. Magnetic Properties

**Figure 3:**
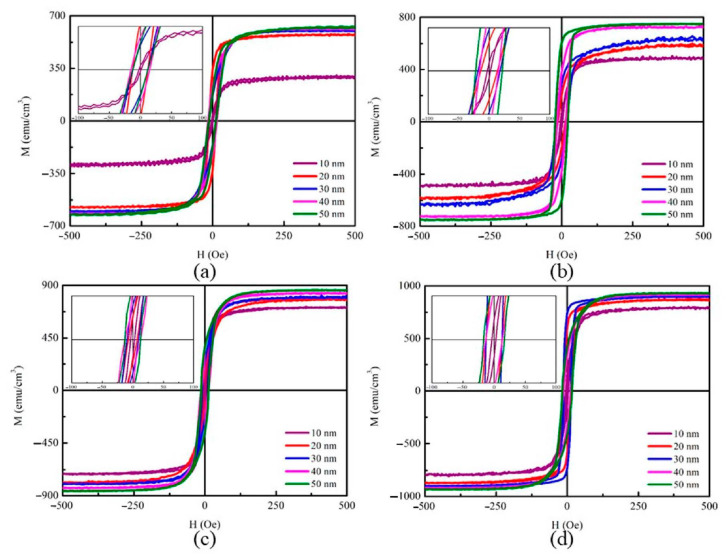
In-plane magnetic hysteresis loop of CoFeBY films. (**a**) RT, (**b**) after annealing at 100 °C, (**c**) after annealing at 200 °C, (**d**) after annealing at 300 °C.

Figure 3a–d plot the in-plane magnetization hysteresis loop of CoFeBY films under the four preparation conditions according to the alternating gradient magnetometry (AGM) measurement.

The plot of the coercivity (Hc) against CoFeBY thickness is presented in the inset. The in-plane magnetic field (H_ext_) of 500 Oe is enough to measure the saturation magnetic spin state. It is important to mention that all samples show no transition into a transitional critical state or rotational anisotropy [30]. 

**Figure 4:**
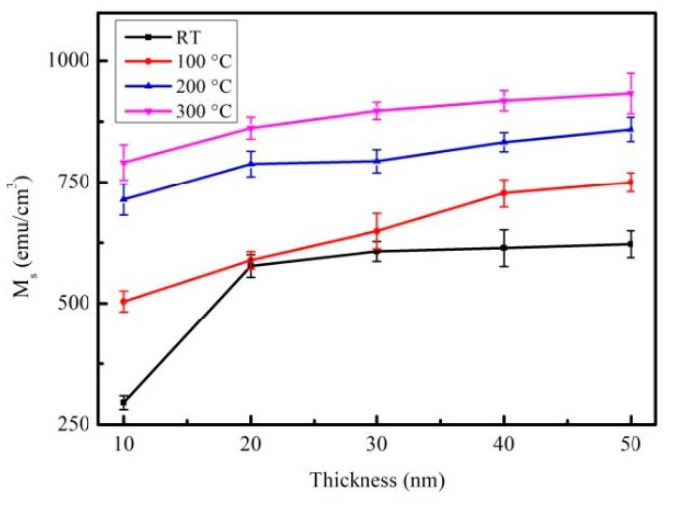
Saturation magnetization (M_S_) of CoFeBY thin films.

The M_S_ of CoFeBY films is shown in Figure 4.

As the film thickness increases from 10 nm to 50 nm, Ms increases. This saturation magnetization depended on thickness, showing the thickness effect of M_S_ in the CoFeBY film. The thin films of as-deposited and post-annealing at 100, 200, and 300 °C had M_S_ in the range of 638 to 933 emu/cm^3^. However, the highest M_S_ of CoFeBY film post-annealing was at 300 °C, indicating it has high spin coupling strength and can induce large M_S_. The addition of Y affects the grain refinement, improves the ferromagnetic spin exchange coupling and causes Ms to enhance the exchange coupling. Nevertheless, oxides may have significant adverse effects on amorphous properties, such as thermal and magnetic properties [31]. The introduction of Y can obtain oxygen and improve the efficiency of the alloy [32,33,34,35]. Therefore, the oxide shown in the previous XRD pattern may be the cause of the increase in magnetic properties.

**Figure 5:**
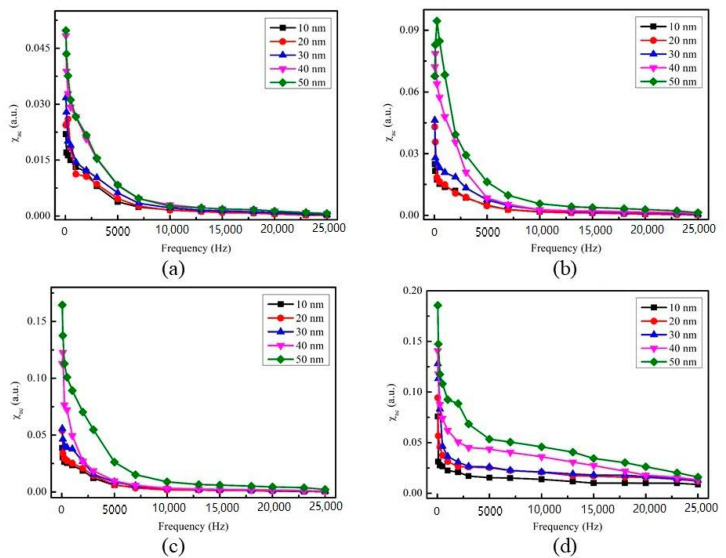
The low-frequency alternate-current magnetic susceptibility (χ_ac_) as a function of the frequency from 50 to 25,000 Hz. (**a**) RT, (**b**) after annealing at 100 °C, (**c**) after annealing at 200 °C, (**d**) after annealing at 300 °C.

Figure 5a–d display the low-frequency alternative-current magnetic susceptibility (χ_ac_) at RT, 100, 200 and 300 °C, under four preparation conditions, with thicknesses ranging from 10 to 50 nm.

In low frequency range of 50–25,000 Hz, the value of χ_ac_ decreases with the increase of frequency. The results also show that when the film thickness is between 10 nm and 50 nm, the corresponding χ_ac_ value increases.

**Figure 6:**
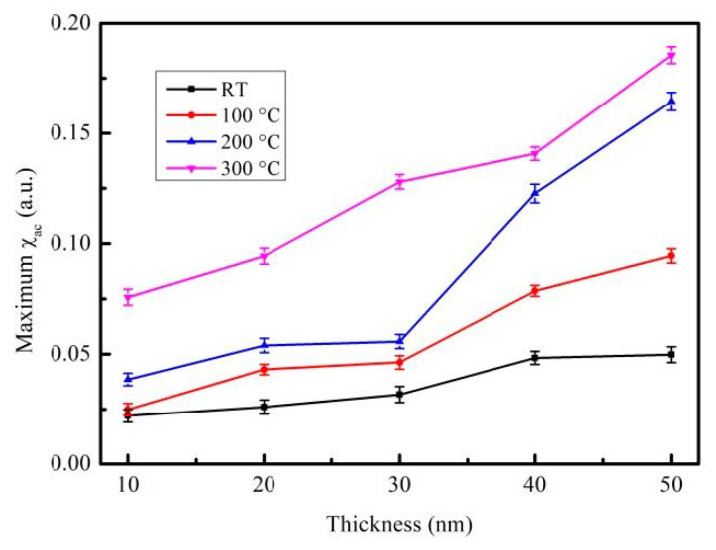
Maximum alternate-current magnetic susceptibility for the CoFeBY films.

The corresponding maximum χ_ac_ with various CoFeBY thicknesses under four preparation conditions are shown in Figure 6. When the maximum χ_ac_ of the CoFeBY film was annealed at 300 °C, the thickness was 50 nm, which is greater than that in other conditions in this investigation. The results showed that the trend of χ_ac_ was consistent with that of M_S_. The addition of Y can enhance the exchange coupling and affect the grain refinement, thus improving the ferromagnetic spin exchange coupling. Table 2 lists the optimal resonance frequency (f_res_) of CoFeBY films under four conditions. The maximum χ_ac_ has the highest spin sensitivity with the optimal resonance frequency [36,37]. The f_res_ value of CoFeBY at various thicknesses is less than 250 Hz. The optimal resonance frequency was estimated to be lower than 500 Hz, thus it can be applied in low-frequency sensors, transformers and magnetic components.

### 3.3. Analysis of Surface Energy and Adhesion

Table 3 depicts contact angles (θ) of the CoFeBY films at RT. The contact angles of the films were measured by using DI water and glycerol. Table 3 displays the contact angles of CoFeBY using DI water (80.8°, 80.7°, 80.1°, 80.2°, and 80.5°), and the contact angles using glycerol (79.6°, 78.0°, 77.7°, 77.4° and 79.3°). Table 4 depicts the contact angles of CoFeBY films that were post-annealed at 100 °C. The contact angles of the films were measured using DI water and glycerol. Table 4 shows the contact angles of CoFeBY using DI water (79.9°, 79.9°, 80.5°, 80.3° and 81.0°), and the contact angles using glycerol (78.1°, 78.1°, 79.7°, 79.3° and 75.1°). Table 5 depicts the contact angles of the CoFeBY films that were post-annealed at 200 °C. The contact angles of the films were detected using DI water and glycerol. Table 5 shows the contact angles of CoFeBY using DI water (78.8°, 79.3°, 78.4°, 78.6° and 78.4°), and the contact angles using glycerol (74.4°, 73.2°, 76.0°, 77.6° and 73.8°). Table 6 depicts the contact angles of the CoFeBY films that were post-annealed at 300 °C. The contact angles of the films were studied using DI water and glycerol. Table 6 shows the contact angles of CoFeBY using DI water (76.6°, 74.0°, 73.0°, 72.0° and 71.7°), and the contact angles using glycerol (75.8°, 72.7°, 73.4°, 71.9° and 71.2°).

The contact angles of the CoFeBY films under all conditions were observed to be less than 90° and the drops were nearly spherical indicating the good hydrophilicity and wettability of the films. Surface energy and adhesion are very important because CoFeBY film can be used as a seed layer or buffer layer. When the surface energy is higher, the liquid absorption is larger and the contact angle decreases. The surface energy is calculated according to the contact angle and Young’s equation [25,26,27].

Young’s Equation (2) is

σ_sg_ = σ_sl_ +σ_lg_ cosθ (2)

In Equation (2), σ_sg_ denotes the surface free energy of the solid, σ_sl_ represents the interfacial tension between liquid and solid, σ_lg_ is the surface tension of the liquid and θ is the contact angle.

**Figure 7:**
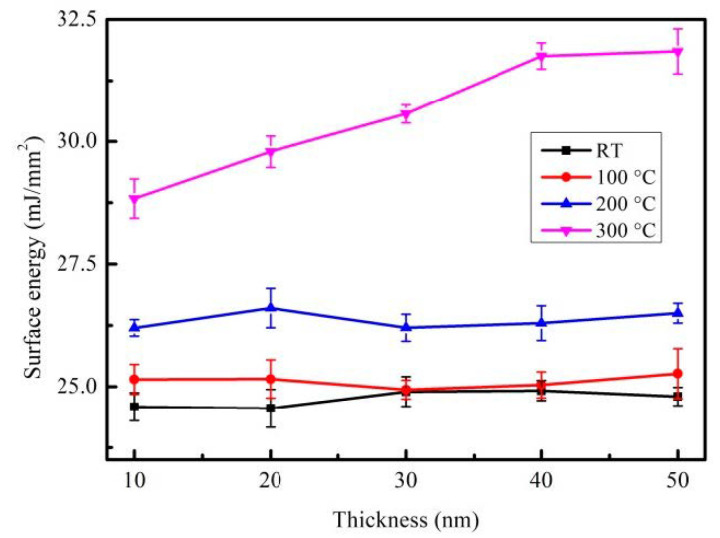
Surface energy of CoFeBY thin films.

Figure 7 shows the surface energy of CoFeBY films under all conditions, including the thickness of CoFeBY films increased from 10 nm to 50 nm at room temperature after annealing at 100, 200 and 300 °C.

It is suggested that the surface energy of post-annealed films was larger than films that were as-deposited. The surface energy ranged from 24.55 to 31.85 mJ/mm^2^. Due to the formation of oxide and oxide layer on the surface of CoFeBY films, the contact angle decreases and the surface energy increases when the samples are exposed to the atmosphere. When the film has a higher surface energy, the adhesion is strongest. The results indicate that it is easier to combine it the layer of the MTJ.

## 4. Conclusions

The structure, magnetism and adhesion of as deposited and annealed CoFeBY films were studied. XRD patterns show that the structure changes from amorphous to crystalline at annealing temperature above 100 °C. The magnetic properties show a thickness effect; as the thickness increased, the induced saturation magnetization of M_S_ and χ_ac_ increased. The addition of Y enhances the exchange coupling, improves the ferromagnetic spin exchange coupling and improves the magnetic properties. As the thickness increases from 10 nm to 50 nm, the surface energy increases. The surface energy of post-annealing film was larger than that of as-deposited film. According to the results of magnetic properties and surface energy, the optimal thickness of CoFeBY film reaches 50 nm after annealing at 300 °C. In the study, it is found that the film is suitable to be used as a free layer of MTJ and can be applied in a MRAM and recording head. The useful properties of this materials can also be estimated for sensor applications and magnetodynamic applications.

## Figures and Tables

**Table 1:** Specific properties for various Glass/CoFeVB, Si (100)/CoFeVB, and Si (100)/CoFeBY materials.

Material	Thickness (nm)	Maximum χ_ac_ (a.u.)	Optimal Resonance Frequency, f_res_ (Hz)	Surface Energy (mJ/mm^2^)
Glass/Co_40_Fe_40_V_10_B_10_ [23]	10–40 at RT	0.068–0.098	50–1000	65.5–38
Si (100)/Co_40_Fe_40_V_10_B_10_ [24]	10–40 at RT	0.013–0.019	50–200	34.2–51.5
Si (100)/Co_40_Fe_40_B_10_Y_10_	10–50 at RT and annealed conditions	0.022–0.185	50–250	24.6–31.9

**Table 2:** Optimal resonance frequency for films of various thicknesses.

Thickness	RT	After Annealing at 100 °C	After Annealing at 200 °C	After Annealing at 300 °C
10 nm	50	50	50	50
20 nm	250	50	50	50
30 nm	50	50	50	50
40 nm	50	100	100	50
50 nm	50	250	50	50

**Table 3:** Average contact angles of CoFeBY thin films at RT with DI water and glycerol.

Co_40_Fe_40_B_10_Y_10_ (10–50 nm)	Contact Angle(θ) with DI Water as Test Liquid	Contact Angle(θ) with Glycerol as Test Liquid
10 nm	80.8°	79.6°
20 nm	80.7°	78.0°
30 nm	80.1°	77.7°
40 nm	80.2°	77.4°
50 nm	80.5°	79.3°

**Table 4:** Average contact angles of CoFeBY thin films after annealing at 100 °C with DI water and glycerol.

Co_40_Fe_40_B_10_Y_10_ (10–50 nm)	Contact Angle(θ) with DI Water as Test Liquid	Contact Angle(θ) with Glycerol as Test Liquid
10 nm	79.9°	78.1°
20 nm	79.9°	78.1°
30 nm	80.5°	79.7°
40 nm	80.3°	79.3°
50 nm	81.0°	75.1°

**Table 5:** Average contact angles of CoFeBY thin films after annealing at 200 °C with DI water and glycerol.

Co_40_Fe_40_B_10_Y_10_ (10–50 nm)	Contact Angle(θ) with DI Water as Test Liquid	Contact Angle(θ) with Glycerol as Test Liquid
10 nm	78.8°	74.4°
20 nm	79.3°	73.2°
30 nm	78.4°	76.0°
40 nm	78.6°	77.6°
50 nm	78.4°	73.8°

**Table 6:** Average contact angles of CoFeBY thin films after annealing at 300 °C with DI water and glycerol.

Co_40_Fe_40_B_10_Y_10_ (10–50 nm)	Contact Angle(θ) with DI Water as Test Liquid	Contact Angle(θ) with Glycerol as Test Liquid
10 nm	76.6°	75.8°
20 nm	74.0°	72.7°
30 nm	73.4°	73.0°
40 nm	72.0°	71.9°
50 nm	71.7°	71.2°

## Data Availability

The data presented in this study are available on request from the corresponding author.
